# Effects of the FXR agonist GW4064 on metabolic disorders in *db/db* mice

**DOI:** 10.1186/s42826-025-00251-9

**Published:** 2026-01-30

**Authors:** Kyuho Kim, Ye-Jee Lee, Jae-Seung Yun, Yu-Bae Ahn, Seung-Hyun Ko

**Affiliations:** Division of Endocrinology and Metabolism, Department of Internal Medicine, College of Medicine, St. Vincent’s Hospital, The Catholic University of Korea, 222 Banpo-daero, Seoul, Seocho-gu 06591 Republic of Korea

**Keywords:** Diabetes mellitus, Endoplasmic reticulum stress, Fatty liver, Insulin resistance, Obesity

## Abstract

**Background:**

Farnesoid X receptor (FXR) is known to play important roles in glucose and lipid metabolism. We aimed to evaluate effects of FXR agonist on metabolic disorders in *db/db* mice. Seven week-old *db/db* mice were injected FXR agonist GW4064 (30 mg/kg/day) or carrier solution (dimethyl sulfoxide) intraperitoneally for 4 weeks. Body weight, food intake, and blood glucose levels were measured weekly. Glucose tolerance test and insulin tolerance test were performed at the end of study. Hepatic genes involed in lipogenesis and gluconeogenesis were analyzed by real time polymerase chain reaction. Endoplasmic reticulum stress markers were analyzed by western blot.

**Results:**

GW4064 treatment significantly attenuated weight gain, and improved glucose intolerance and insulin resistance in *db/db* mice. In addition, GW4064 treatment significantly repressed hepatic steatosis. GW4064 treatment significantly lowered hepatic gene expression of *phosphoenolpyruvate carboxykinase 1*, *glucose 6-phosphatase*, and *sterol regulatory element binding protein 1c*. GW4064 treatment significantly lowered the protein levels of ATF6, CHOP, Caspase3, and Cleaved Caspase3 in liver. FXR agonist GW4064 showed beneficial effects on weight gain, glucose intolerance, insulin resistance, hepatic steatosis, and hepatic ER stress.

**Conclusions:**

These findings suggest that FXR agonists are promising therapeutic agents for treatment of various metabolic disorders.

**Supplementary Information:**

The online version contains supplementary material available at 10.1186/s42826-025-00251-9.

## Background

The Farnesoid X receptor (FXR) is a member of the nuclear hormone receptor superfamily [1]. It is highly expressed in the liver, intestine, kidney, and adrenal gland [2]. FXR is involved in maintaining bile acid homeostasis by controlling catabolism of cholesterol to bile acids and secretion of bile acids into the bile [3, 4]. In addition, FXR plays an important role in glucose and lipid metabolism, and FXR^−/−^ mice exhibited glucose intolerance and insulin resistance [5]. Activation of FXR improved hyperglycemia by repressing hepatic gluconeogenesis and increasing hepatic glycogen synthesis [6, 7]. Activation of FXR lowered plasma triglyceride levels by repressing the expression of hepatic *sterol regulatory element binding protein 1c (SREBP1c)* [8] and enhancing plasma triglyceride clearance [9, 10]. In addition, previous studies demonstrated that FXR activation prevents diet-induced hepatic steatosis and fibrosis [11, 12].

Metabolic disorders such as hyperglycemia, insulin resistance, dyslipidemia, obesity, and hypertension tend to cluster together [13]. Leptin receptor-deficient *db/db* mice are a well validated mouse model for the study of metabolic disorders [14], and an excellent mouse model for examining the effects of new therapeutic metabolic agents [15].

In this study, we examined the effects of the FXR agonist GW4064 on the metabolic disorders of obesity, glucose intolerance, insulin resistance, dyslipidemia, and hepatic steatosis in *db/db* mice. We also investigated the role of GW4064 in hepatic endoplasmic reticulum (ER) stress.

## Methods

### Animals experiments

Animal protocols were approved by the Laboratory Animal Care Committee at The Catholic University of Korea (2019–0221–03). Seven-week-old male C57BLKS/J *db/db* mice were purchased from The Jackson laboratory (Bar Harbor, ME, USA). Mice were housed under standard conditions with a 12-h light, 12-h dark cycle in climate-controlled, pathogen-free barrier facilities, and a standard chow diet and water were provided ad libitum. We randomly divided *db/db* mice into 2 groups (*n* = 5 per group). GW4064 (30 mg/kg) or carrier solution (dimethyl sulfoxide [DMSO]) was administered intraperitoneally once a day for 4 weeks. Body weight and food intake were measured weekly. The blood samples were obtained from tail veins after overnight fasting every week, and glucose concentrations were measured using an Accu-check glucometer (Roche, Basel, Switzerland). For the glucose tolerance tests, mice fasted for 12 h before an intraperitoneal injection (2 g/kg body weight) of D-glucose in deionized water. For the insulin tolerance tests, mice fasted for six hours before an intraperitoneal injection (1 U/kg body weight) of Humalog (Eli Lilly and Company, Indianapolis, IN, USA). All animals were euthanized at 11 weeks of age.

### Histochemical analysis

After mice were sacrificed, liver tissues were collected and fixed in 10% neutral buffered formalin for six hours at 4° C and then washed with deionized water. After processing using an automatic tissue processor, the samples were embedded in paraffin. Tissue blocks were sectioned at 5 μm thickness and were mounted on adhesive slides. H & E staining was performed using an H & E staining kit (Abcam, Ann Arbor, MI, USA).

### Analysis of serum lipids and insulin levels

Blood was collected via cardiac puncture from mice after overnight fasting. Serum levels of total cholesterol, and triglycerides were measured at Neodin VetLab (Guri, Korea) using a chemistry analyzer BS-400 (Mindray, Shenzhen, China). Serum insulin levels were measured using an insulin ELISA kit (ALPCO, Salem, NH, USA). The homeostasis model for insulin resistance (HOMA-IR) was calculated from the fasting blood glucose (mg/dl) × fasting serum insulin (μU/ml) devided by 405.

### Real-time PCR analysis

Total RNA was purified from the mouse liver using RiboEx (GeneAll, Seoul, Korea). Superscript III reverse transcriptase (Invitrogen, Carlsbad, CA, USA) was used to synthesize 1 μg of cDNA in total RNA. A 20 ml reaction mixture was prepared containing cDNA and TB Green Premix Ex Taq (Takara Bio, Shiga, Japan) and 10 pmol/ml primer pairs: *small heterodimer partner (SHP)* forward (CTTCAACCCAGATGTGCCAG), *SHP* reverse (GAGGCCATGAGGA GGATTCG), *FXR* forward (GCTTCCAGGGTTTCAGACAC), *FXR* reverse (CTTTCTTCCA ACAGGTCTGC), *phosphoenolpyruvate carboxykinase 1 (PEPCK1)* forward (AGCATATGCTGATCCTGGGC), *PEPCK1* reverse (CTTAAGTTGCCTTGGGCAT C), *glucose 6-phosphatase (G6Pase)* forward (GGATTCCGGTGTTTGAACG), *G6Pase* reverse (GCAATGCCTGACAAGACTCC), *SREBP1c* forward (CTGCATGCCATGGGCAA GTA), *SREBP1c* reverse (GCTCAGGAAGAAACGTGTCAAG), *28S rRNA* forward (GATTC CCACTGTCCCTACC), and *28S rRNA* reverse (ACCTCTCATGTCTCTTCACC). The reaction mixture was measured and analyzed by real-time PCR (Bio-Rad, Hercules, CA, USA). The mRNA levels of target genes were normalized to that of 28S rRNA.

### Western blot analysis

Protein was extracted from the mouse liver using a protein extraction solution (iNtRON Biotechnology, Seongnam, Korea). Proteins were collected by centrifugation at 13,000 rpm for 20 min. The protein level was quantified by the BCA method. Twenty micrograms of total proteins were separated by 10% SDS-PAGE gels and transferred to polyvinylidene fluoride membranes. Membranes were blocked in a Tris-buffered solution containing 5% skim milk (BD) for one hour at room temperature. Immunoblotting was performed at 4℃ overnight with primary antibodies of activating transcription factor 6 (ATF6) (1:1000, ABclonal, Woburn, MA, USA), C/EBP homologous protein (CHOP) (1:1000, ABclonal, Woburn, MA, USA), Caspase3 (1:1000, Santa Cruz, CA; Dallas, TX, USA), Cleaved Caspase3 (1:1000, Cell Signaling Technology, Boston, MA, USA), and β-actin (1:5000, Abcam, Waltham, MA, USA). After being washed, the membrane was incubated in a 1:3000 dilution of secondary antibody (anti-mouse, 1:10,000 and anti-rabbit, 1:10,000, GenDEPOT, Baker, TX, USA) at room temperature for one hour. We used the ECL Select Western blotting detection reagent (GE Healthcare, Lafayette, IN, USA). The band was identified using an Image Analyser system (Syngene, Cambridge, UK) and quantified using Multi Gauge V3.0 software.

### Statistical analysis

Data are presented as mean ± standard error of the mean (SEM). Statistical significance was determined by Student’s t-test. *P* < 0.05 was considered statistically significant.

## Results

### FXR agonist GW4064 attenuated weight gain

**Fig. 1:**
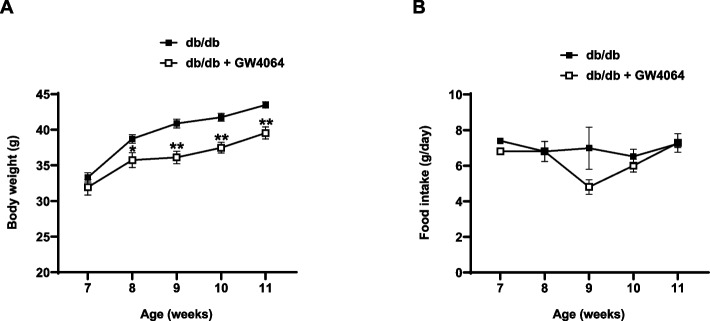
The FXR agonist GW4064 attenuated weight gain. **7**-week-old male *db/db* mice were intraperitoneally injected with GW4064 (30 mg/kg/day, *n* = 5) or carrier solution (dimethyl sulfoxide [DMSO], *n* = 5) as the control for 4 weeks. **A** Growth curve. **B** Food intake. The data are presented as the mean ± SEM. *P < 0.05, **P < 0.01 compared to control

7-week-old male *db/db* mice were treated with GW4064 (30 mg/kg, daily) or vehicle for 4 weeks. As expected, *db/db* mice exhibited a progressive increase in body weight. GW4064 treatment significantly attenuated the increase in weight gain (Fig. 1A). A statistical difference was confirmed as early as the 1 st weeks of GW4064 treatment, and the difference in body weight was 3.9 g between the groups at the end of the experiment. Food intake was comparable between the two groups (Fig. 1B).

### FXR agonist GW4064 improved glucose intolerance

**Fig. 2:**
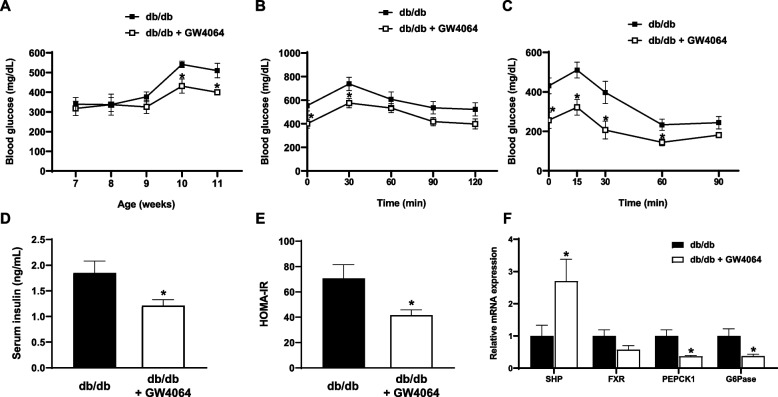
The FXR agonist GW4064 improved glucose intolerance. **A** Fasting glucose. **B** Blood glucose levels after intraperitoneal injection of glucose (2 g/kg body weight) after 4 weeks of GW4064 treatment. **C** Blood glucose levels after intraperitoneal injection of insulin lispro (0.75 U/kg body weight) after 4 weeks of treatment with GW4064. **D** Serum insulin levels after 4 weeks of treatment. **E** HOMA-IR values after 4 weeks of treatment. **F** Relative mRNA expression of SHP, FXR, PEPCK1, and G6Pase in the liver after 4 weeks of treatment. The data are presented as the mean ± SEM. *P < 0.05 compared to control

GW4064 treatment significantly reduced fasting plasma glucose levels after 3 weeks of treatment (Fig. 2A). Results of glucose tolerance tests showed an improved glucose intolerance in GW4064-treated mice (Fig. 2B). In addition, results of insulin tolerance tests showed an improved insulin resistance in GW4064-treated mice (Fig. 2C). Serum insulin levels and homeostasis model assessment of insulin resistance were significantly lower in GW4064-treated mice (Fig. 2D, E). GW4064 treatment significantly increased hepatic gene expression of *SHP* and significantly lowered hepatic gene expression *PEPCK1* and *G6Pase*, the two important hepatic gluconeogenic genes (Fig. 2F).

### GW4064 repressed hepatic steatosis

**Fig. 3:**
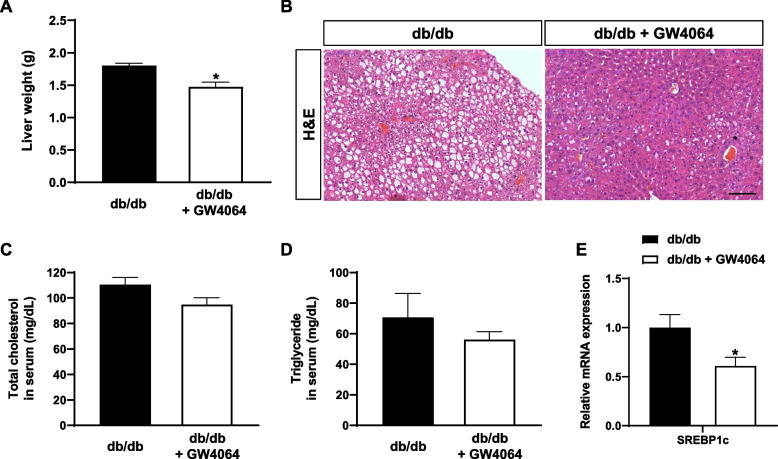
The FXR agonist GW4064 repressed hepatic steatosis. At the end of the 4-week treatment, mice were sacrificed, and livers were harvested. **A** Liver weights. **B** Representative images of H & E staining of the liver. Scale bar = 100 μm. **C** Serum levels of total cholesterol. **D** Serum levels of triglycerides. **E** Relative mRNA expression of SREBP1c in the liver. The data are presented as the mean ± SEM. *P < 0.05 compared to control

To examine hepatic lipid accumulation, liver weight was measured after mice were sacrificed at the end of treatment. The average liver weight of control mice was 1.8 g compared to 1.5 g in GW4064-treated mice, and there was significant difference between the two groups (Fig. 3A). H & E staining of liver sections showed significantly reduced hepatic lipid accumulation in GW4064-treated mice (Fig. 3B). There was no statistical difference in serum levels of total cholesterol and triglycerides between the two groups (Fig. 3C, D). GW4064 treatment significantly lowered hepatic gene expression of *SREBP1c* (Fig. 3E).

### GW4064 decreased ER stress-induced apoptosis in the liver

**Fig. 4:**
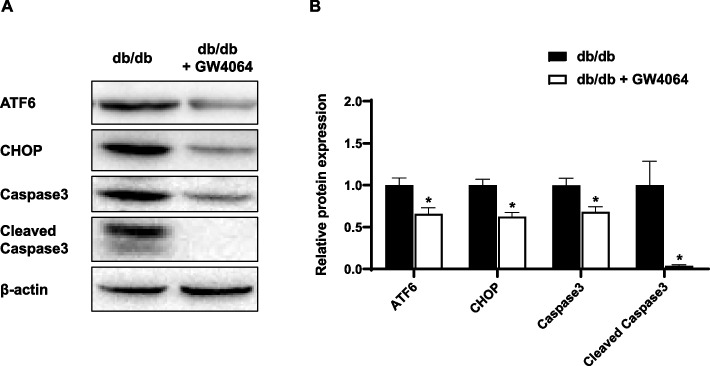
The FXR agonist GW4064 decreased ER stress-induced apoptosis in the liver. At the end of the 4-week treatment, mice were sacrificed, and livers were harvested. **A** Protein expression levels of ATF6, CHOP, Caspase3, and Cleaved Caspase3 in the liver. **B** Relative protein expression of ATF6, CHOP, Caspase3, and Cleaved Caspas3 in the liver. The data are presented as the mean ± SEM. *P < 0.05 compared to control

GW4064 treatment significantly decreased protein levels of ATF6 and CHOP in the liver. Western blot showed that Caspase3 and Cleaved Caspase3 expression, related to apoptosis of the liver, decreased in GW4064-treated mice (Fig. 4).

## Discussion

The current study demonstrated that the FXR agonist GW4064 suppressed body weight gain, improved glucose intolerance, and reduced hepatic steatosis in *db/db* mice. GW4064 treatment reduced the expression of genes involved in hepatic gluconeogenesis and lipogenesis and decreased ER stress and ER stress-related apoptosis in the liver. Thus, the FXR agonist GW4064 showed beneficial effects on various metabolic disorders. This study suggests that FXR can be a promising therapeutic option for treatment of various metabolic disorders.

This study found that 4 weeks of intraperitoneal treatment with GW4064 (30 mg/kg/day) suppressed weight gain in *db/db* mice. This result is in line with a previous study that demonstrated suppression of weight gain in high fat diet (HFD)-fed mice after 6 weeks of intraperitoneal treatment with GW4064 (50 mg/kg/day) [11]. We considered that a potential mechanism of GW4064-induced weight loss is the induction of fibroblast growth factor 15 (FGF15) through intestinal FXR activation, which promotes the expression of genes involved in thermogenesis, mitochondrial biogenesis, and fatty acid oxidation, eventually leading to an increased metabolic rate [16, 17]. However, another study demonstrated no effect of GW4064 on weight gain in *db/db* mice after five days of oral gavage with GW4064 (30 mg/kg, twice a day) [6]. Another study showed accentuation of weight gain in HFD-fed mice after three months of oral administration of GW4064 (15 mg/kg/day) [18]. We assumed that the different routes of administration of GW4064 might induce different results in terms of weight gain.

GW4064 treatment improved hyperglycemia in *db/db* mice. Improved hyperglycemia can be explained by lower mRNA levels of *PEPCK1* and *G6Pase* in the liver of GW4064-treated mice, in agreement with results of previous studies [6, 11]. Reduction of hepatic lipid accumulation and subsequent improved insulin resistance also can improve hyperglycemia [19]. In addition, FXR activation can protect pancreatic islets from lipotoxicity and improve the stimulation index [20], enhance thermogenesis in brown adipose tissue and the browning of white adipose tissue [17, 21], and increase skeletal muscle mass and improve muscle performance [22]. These effects across various organs may contribute to the improvement of hyperglycemia.

FXR activation can control lipid metabolism in various ways. GW4064 treatment can suppress expression of *SREBP1c* and inhibit lipogenesis, in agreement with results in our study [18]. Activation of FXR can enhance peroxisome proliferator activated receptor alpha (PPARα) activity and increase triglyceride clearance [23]. In addition, GW4064 treatment can suppress expression of the fatty acid transporter CD36 [11]. Previous studies have demonstrated a significant decrease in serum levels of total cholesterol and triglycerides following GW4064 treatment compared with controls [11, 24]. However, in our study, although we observed a trend toward reduced serum cholesterol and triglyceride levels after GW4064 treatment, the changes were not statistically significant. We assume that the relatively short duration of GW4064 treatment in this study was insufficient to produce a significant effect.

In this study, GW4064 treatment attenuated ER stress and ER stress-related apoptosis in the liver. This is in line with a previous study that demonstrated that activation of FXR inhibited ER stress-induced NLR family pyrin domain containing 3 (NLRP3) inflammasome activation in hepatocytes, attenuating ER stress-induced hepatocyte death and liver injury [25]. These results suggest that FXR is a promising target for the treatment of metabolic dysfunction-associated steatotic liver disease. Clinical trials of synthetic FXR agonists such as obeticholic acid [26] or cilofexor [27] demonstrated demonstrated supporting results.

## Conclusions

The FXR agonist GW4064 showed beneficial effects on various metabolic disorders such as obesity, glucose intolerance, insulin resistance, and hepatic steatosis. Study findings suggest that pharmacological activation of FXR can be a promising therapeutic option for treatment of various metabolic disorders.

## Supplementary Information

Supplementary material 1.

## Data Availability

The data and materials supporting the fndings of this study are available from the corresponding author upon reasonable request.

## Abbreviations

ATF6: Activating transcription factor 6
CHOP: C/EBP homologous protein
DMSO: Dimethyl sulfoxide
ER: Endoplasmic reticulum
FXR: Farnesoid X receptor
G6Pase: Glucose 6-phosphatase
HFD: High fat diet
HOMA-IR: Homeostasis model for insulin resistance
NLRP3: NLR family pyrin domain containing 3
PEPCK1: Phosphoenolpyruvate carboxykinase 1
SEM: Standard error of the mean
SHP: Small heterodimer partner
SREBP1c: Sterol regulatory element binding protein 1c

## References

[1] Sun L, Cai J, Gonzalez FJ. The role of farnesoid X receptor in metabolic diseases, and gastrointestinal and liver cancer. Nat Rev Gastroenterol Hepatol. 2021;18(5):335–47. 33568795 10.1038/s41575-020-00404-2

[2] Lu TT, Repa JJ, Mangelsdorf DJ. Orphan nuclear receptors as eLiXiRs and FiXeRs of sterol metabolism. J Biol Chem. 2001;276(41):37735–8. 11459853 10.1074/jbc.R100035200

[3] Sinal CJ, Tohkin M, Miyata M, Ward JM, Lambert G, Gonzalez FJ. Targeted disruption of the nuclear receptor FXR/BAR impairs bile acid and lipid homeostasis. Cell. 2000;102(6):731–44. 11030617 10.1016/s0092-8674(00)00062-3

[4] Kok T, Hulzebos CV, Wolters H, Havinga R, Agellon LB, Stellaard F, et al. Enterohepatic circulation of bile salts in farnesoid X receptor-deficient mice: efficient intestinal bile salt absorption in the absence of ileal bile acid-binding protein. J Biol Chem. 2003;278(43):41930–7. 12917447 10.1074/jbc.M306309200

[5] Ma K, Saha PK, Chan L, Moore DD. Farnesoid x receptor is essential for normal glucose homeostasis. J Clin Invest. 2006;116(4):1102–9. 16557297 10.1172/JCI25604 PMC1409738

[6] Zhang Y, Lee FY, Barrera G, Lee H, Vales C, Gonzalez FJ, et al. Activation of the nuclear receptor FXR improves hyperglycemia and hyperlipidemia in diabetic mice. Proc Natl Acad Sci U S A. 2006;103(4):1006–11. 16410358 10.1073/pnas.0506982103 PMC1347977

[7] Cariou B, van Harmelen K, Duran-Sandoval D, van Dijk TH, Grefhorst A, Abdelkarim M, et al. The farnesoid X receptor modulates adiposity and peripheral insulin sensitivity in mice. J Biol Chem. 2006;281(16):11039–49. 16446356 10.1074/jbc.M510258200

[8] Watanabe M, Houten SM, Wang L, Moschetta A, Mangelsdorf DJ, Heyman RA, et al. Bile acids lower triglyceride levels via a pathway involving FXR, SHP, and SREBP-1c. J Clin Invest. 2004;113(10):1408–18. 15146238 10.1172/JCI21025 PMC406532

[9] Kast HR, Nguyen CM, Sinal CJ, Jones SA, Laffitte BA, Reue K, et al. Farnesoid X-activated receptor induces apolipoprotein C-II transcription: a molecular mechanism linking plasma triglyceride levels to bile acids. Mol Endocrinol. 2001;15(10):1720–8. 11579204 10.1210/mend.15.10.0712

[10] Claudel T, Inoue Y, Barbier O, Duran-Sandoval D, Kosykh V, Fruchart J, et al. Farnesoid X receptor agonists suppress hepatic apolipoprotein CIII expression. Gastroenterology. 2003;125(2):544–55. 12891557 10.1016/s0016-5085(03)00896-5

[11] Ma Y, Huang Y, Yan L, Gao M, Liu D. Synthetic FXR agonist GW4064 prevents diet-induced hepatic steatosis and insulin resistance. Pharm Res. 2013;30(5):1447–57. 23371517 10.1007/s11095-013-0986-7 PMC3664363

[12] Cipriani S, Mencarelli A, Palladino G, Fiorucci S. FXR activation reverses insulin resistance and lipid abnormalities and protects against liver steatosis in Zucker (fa/fa) obese rats. J Lipid Res. 2010;51(4):771–84. 19783811 10.1194/jlr.M001602 PMC2842143

[13] Cornier MA, Dabelea D, Hernandez TL, Lindstrom RC, Steig AJ, Stob NR, et al. The metabolic syndrome. Endocr Rev. 2008;29(7):777–822. 18971485 10.1210/er.2008-0024 PMC5393149

[14] Coleman DL. Obese and diabetes: two mutant genes causing diabetes-obesity syndromes in mice. Diabetologia. 1978;14(3):141–8. 350680 10.1007/BF00429772

[15] Daniels Gatward LF, Kennard MR, Smith LIF, King AJF. The use of mice in diabetes research: the impact of physiological characteristics, choice of model and husbandry practices. Diabet Med. 2021;38(12):e14711. 34614258 10.1111/dme.14711

[16] Fu L, John LM, Adams SH, Yu XX, Tomlinson E, Renz M, et al. Fibroblast growth factor 19 increases metabolic rate and reverses dietary and leptin-deficient diabetes. Endocrinology. 2004;145(6):2594–603. 14976145 10.1210/en.2003-1671

[17] Fang S, Suh JM, Reilly SM, Yu E, Osborn O, Lackey D, et al. Intestinal FXR agonism promotes adipose tissue browning and reduces obesity and insulin resistance. Nat Med. 2015;21(2):159–65. 25559344 10.1038/nm.3760 PMC4320010

[18] Watanabe M, Horai Y, Houten SM, Morimoto K, Sugizaki T, Arita E, et al. Lowering bile acid pool size with a synthetic farnesoid X receptor (FXR) agonist induces obesity and diabetes through reduced energy expenditure. J Biol Chem. 2011;286(30):26913–20. 21632533 10.1074/jbc.M111.248203 PMC3143650

[19] Perry RJ, Samuel VT, Petersen KF, Shulman GI. The role of hepatic lipids in hepatic insulin resistance and type 2 diabetes. Nature. 2014;510(7503):84–91. 24899308 10.1038/nature13478 PMC4489847

[20] Popescu IR, Helleboid-Chapman A, Lucas A, Vandewalle B, Dumont J, Bouchaert E, et al. The nuclear receptor FXR is expressed in pancreatic beta-cells and protects human islets from lipotoxicity. FEBS Lett. 2010;584(13):2845–51. 20447400 10.1016/j.febslet.2010.04.068

[21] Kim SH, Park WY, Kim B, Kim JH, Song G, Park JY, et al. FXR-ApoC2 pathway activates UCP1-mediated thermogenesis by promoting the browning of white adipose tissues. J Biol Chem. 2025;301(3):108181. 39798876 10.1016/j.jbc.2025.108181 PMC11871442

[22] Qiu Y, Yu J, Ji X, Yu H, Xue M, Zhang F, et al. Ileal FXR-FGF15/19 signaling activation improves skeletal muscle loss in aged mice. Mech Ageing Dev. 2022;202:111630. 35026209 10.1016/j.mad.2022.111630

[23] Pineda Torra I, Claudel T, Duval C, Kosykh V, Fruchart JC, Staels B. Bile acids induce the expression of the human peroxisome proliferator-activated receptor alpha gene via activation of the farnesoid X receptor. Mol Endocrinol. 2003;17(2):259–72. 12554753 10.1210/me.2002-0120

[24] Han SY, Song HK, Cha JJ, Han JY, Kang YS, Cha DR. Farnesoid X receptor (FXR) agonist ameliorates systemic insulin resistance, dysregulation of lipid metabolism, and alterations of various organs in a type 2 diabetic kidney animal model. Acta Diabetol. 2021;58(4):495–503. 33399988 10.1007/s00592-020-01652-z

[25] Han CY, Rho HS, Kim A, Kim TH, Jang K, Jun DW, et al. FXR inhibits endoplasmic reticulum stress-induced NLRP3 inflammasome in hepatocytes and ameliorates liver injury. Cell Rep. 2018;24(11):2985–99. 30208322 10.1016/j.celrep.2018.07.068

[26] Younossi ZM, Ratziu V, Loomba R, Rinella M, Anstee QM, Goodman Z, et al. Obeticholic acid for the treatment of non-alcoholic steatohepatitis: interim analysis from a multicentre, randomised, placebo-controlled phase 3 trial. Lancet. 2019;394(10215):2184–96. 31813633 10.1016/S0140-6736(19)33041-7

[27] Patel K, Harrison SA, Elkhashab M, Trotter JF, Herring R, Rojter SE, et al. Cilofexor, a nonsteroidal FXR agonist, in patients with noncirrhotic NASH: a phase 2 randomized controlled trial. Hepatology. 2020;72(1):58–71. 32115759 10.1002/hep.31205

