# Supplementary material for: Effects of the FXR agonist GW4064 on metabolic disorders in db/db mice
Source: Lab Anim Res. 2026 Jan 30;42:5. doi: 10.1186/s42826-025-00251-9 (PMC12857054; doi:10.1186/s42826-025-00251-9)
Supplement: Supplementary file 1 — Supplementary material 1. [file 42826_2025_251_MOESM1_ESM.docx]

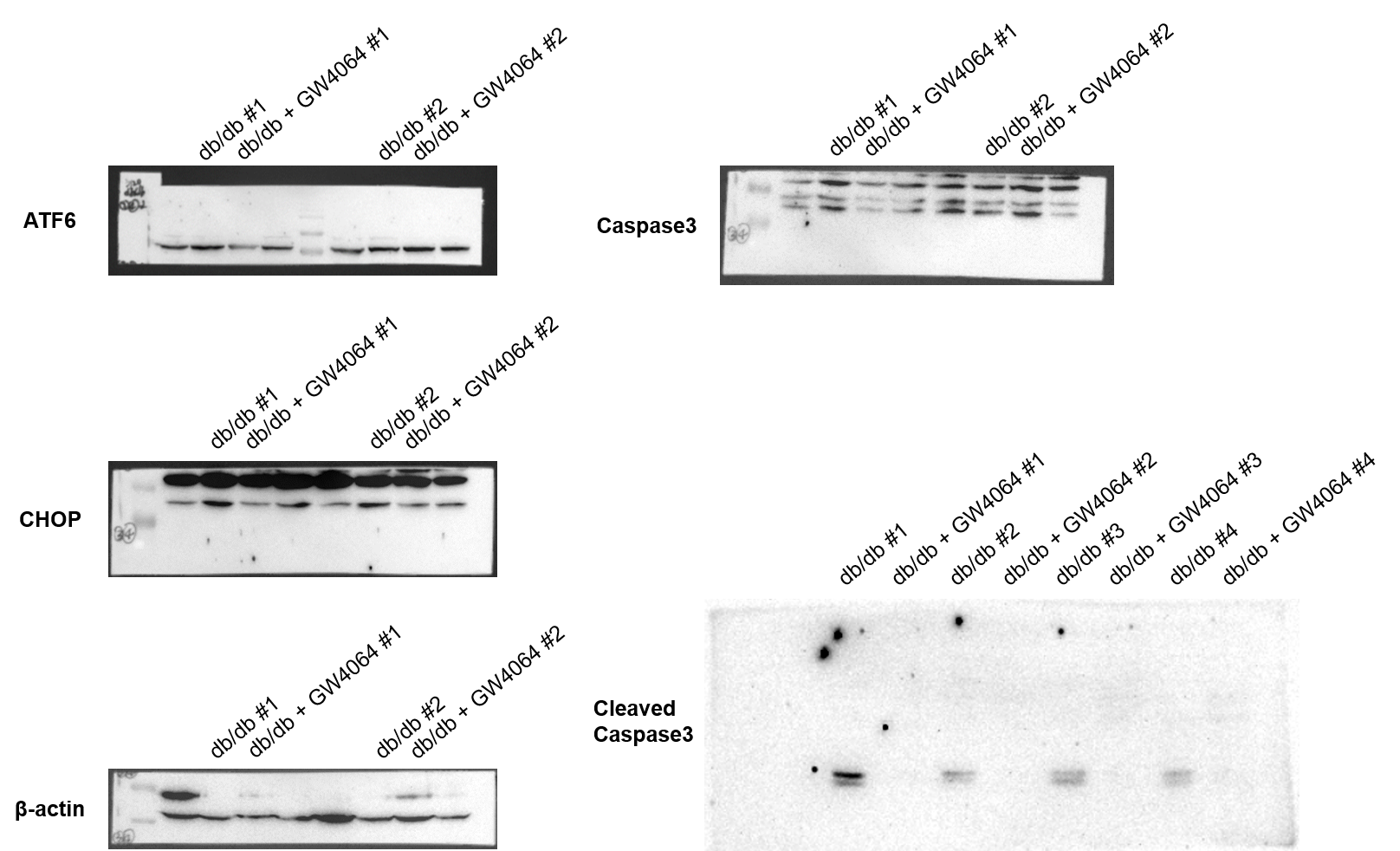


**Fig. 4 The FXR agonist GW4064 decreased ER stress-induced apoptosis in the liver.** At the end of the 4-week treatment, mice were sacrificed, and livers were harvested. (A) Protein expression levels of ATF6, CHOP, Caspase3, and Cleaved Caspase3 in the liver. (B) Relative protein expression of ATF6, CHOP, Caspase3, and Cleaved Caspas3 in the liver.
